# Calcium and zinc status and risk of preeclampsia in pregnant women with varying seafood intake in Ambon City: a case-control study

**DOI:** 10.1186/s13104-026-07662-4

**Published:** 2026-04-28

**Authors:** Merlin Margreth Maelissa, Anita Deborah Anwar, Johanes Cornelius Mose, Sofie Rifayani Krisnadi, Marliyati Sanaky, Arlen Resnawaldi, Udin Sabarudin, Akhmad Yogi Pramatirta, Adhi Pribadi

**Affiliations:** 1 Department of Obstetrics and Gynecology, Faculty of Medicine, Pattimura University, Ambon, 97233 Indonesia; 2 Maternal Fetal Medicine Division, Department of Obstetrics and Gynecology, Faculty of Medicine, Universitas Padjadjaran; Dr. Hasan Sadikin General Hospital, Bandung, 40161 Indonesia

**Keywords:** Preeclampsia, Micronutrient deficiency, Marine diet in pregnancy

## Abstract

**Background:**

Preeclampsia is a significant cause of maternal morbidity and mortality, often influenced by maternal nutrition. Calcium and zinc are essential micronutrients implicated in the prevention of preeclampsia. This study examines their levels in pregnant women consuming seafood in Ambon City as a local data that could support global data.

**Methods:**

This analytical observational study used a case-control design. Calcium and zinc levels were assessed in 120 pregnant women (60 with preeclampsia, 60 without preeclampsia) attending medical facilities in Ambon from June to October 2024. Statistical analyses, including Chi-square tests, were performed. A P-value < 0.05 was considered statistically significant.

**Results:**

Pregnant women with preeclampsia in Ambon City had lower calcium levels than those without preeclampsia, especially among those consuming less than four pieces of sea fish per day (≈ 120 g each) (borderline (OR = 3.143, *p* = 0.053)). Zinc levels showed no significant difference. Women with preeclampsia had a 10.286-fold increased likelihood of low calcium levels.

**Conclusion:**

In Ambon City, pregnant women with preeclampsia have lower calcium levels than those without, while zinc levels remain similar between both groups. Higher fish consumption (at least four medium-sized pieces per day) is linked to higher calcium levels in both preeclamptic and non-preeclamptic women, but zinc levels are unaffected by fish intake.

## Introduction

Preeclampsia (PE) is a multisystem disorder that occurs after 20 weeks of gestation. It impacts pregnancy in various ways beyond hypertension, including proteinuria, liver dysfunction, placental abruption, and intrauterine growth restriction. As a subtype of hypertensive disorders of pregnancy, it complicates approximately 5% of pregnancies worldwide. Preeclampsia remains a leading cause of maternal, fetal, and neonatal mortality, particularly in low- and middle-income countries. Despite extensive research, the etiology of preeclampsia remains largely elusive [1].

Globally, preeclampsia and eclampsia account for around 63,000 maternal deaths annually. Besides maternal mortality and morbidity, preeclampsia contributes to 500,000 infant deaths each year [2]. In Indonesia, the prevalence of pre clampsia is approximately 120,000 cases annually, affecting about 5% of all pregnancies. Reports from the Maluku Provincial Health Office in 2021 indicated a maternal mortality rate of 114 per 100,000 live births, with 70 maternal deaths primarily caused by hypertensive disorders in pregnancy and postpartum hemorrhage [3]. Between 2018 and 2021, Dr. M. Haulussy Regional Hospital in Ambon City identified preeclampsia-eclampsia as the leading cause of maternal mortality, accounting for 20.41% of cases. At Dr. Johannes Leimena Regional Hospital, preeclampsia accounted for 10.22% of cases in 2023.

Studies have established the significant role of micronutrients in pregnancy and fetal development. Imbalances in micronutrient levels can disrupt maternal and fetal metabolism. As components of enzymes and transcription factors, micronutrients are essential for growth and development. Proper implantation and angiogenesis are critical for maintaining a healthy pregnancy. Deficiencies in elements such as calcium, magnesium, zinc, and copper have been linked to an increased risk of preeclampsia. These micronutrients are vital to the antioxidant defense system, and an imbalance between oxidative and antioxidant systems can lead to oxidative stress, a key factor in endothelial dysfunction and the pathogenesis of preeclampsia [4, 5].

Zinc is the second most abundant trace element after iron and is crucial for all living organisms. It functions as a divalent and redox-inactive cation, which explains its diverse physiological roles across biological processes. Zinc is an integral part of many systems, including DNA polymerase complexes. Its deficiency has been associated with conditions such as preeclampsia, various cancers, cardiovascular diseases, immune dysfunction, aging, and infections. During pregnancy, zinc is critical for normal development and fetal growth. Deficiency during pregnancy or early childhood can lead to stunted growth, mental retardation, and delayed sexual maturation. The role of zinc in preeclampsia remains poorly understood, but it is hypothesized that zinc deficiency increases lipid peroxidation levels, contributing to oxidative stress—a mechanism involved in preeclampsia. Furthermore, zinc acts as a cofactor for antioxidant enzymes, highlighting its importance in maintaining oxidative balance [6, 7].

Low calcium levels during pregnancy are associated with increased blood pressure. Pregnant women with high calcium intake typically have stable blood pressure, reducing the risk of preeclampsia. Calcium deficiency can lead to elevated parathyroid hormone levels, increasing intracellular calcium. This elevation causes vascular smooth muscle vasoconstriction, resulting in hypertension—a risk factor for preeclampsia. In preeclampsia, reduced extracellular calcium levels and low ionized serum calcium concentrations disrupt endothelial protein synthesis, including prostacyclin and nitric oxide (NO). Consequently, calcium deficiency contributes not only to mineral deficiencies but also to pathological effects induced by oxidative stress [6–9]. Between 2011 and 2020, the World Health Organization (WHO) published and updated guidelines recommending prenatal calcium supplementation to prevent preeclampsia and its complications. The available evidence consistently supports daily calcium supplementation (1.5–2.0 g elemental calcium) for pregnant women with low dietary calcium intake. In 2020, WHO extended this recommendation to include preconception calcium supplementation for populations at risk [10].

Seafood, particularly fish, plays a crucial role in global food and nutritional security. Fish is a rich source of essential nutrients, including bioavailable proteins, long-chain omega-3 fatty acids, and micronutrients such as vitamins A, B12, and D, zinc, calcium, selenium, and iodine [11, 12]. In Maluku, fish is a primary source of protein, vitamins, and minerals. Commonly consumed species include skipjack tuna, yellowfin tuna, mackerel tuna, scad, anchovies, and sardines. These species are abundant in Ambon City waters and readily available at local markets and restaurants, ensuring widespread access to this nutrient-dense food source [11–14].

Ambon’s dietary pattern is unique compared to other regions of Indonesia, with seafood, particularly fish, being consumed daily in significantly higher quantities. This distinctive nutritional environment justifies Ambon as a valuable setting for studying the relationship between micronutrient intake and preeclampsia. Based on regional dietary surveys, the threshold of “≥4 pieces of fish per day” has been established, which corresponds to approximately 480 g/day, assuming one piece equals about 120 g of fish. This conversion provides an objective basis for assessing fish consumption in the present study [11–14].

## Methods

### Study design and eligibility

This study employed an analytical observational design with a case-control approach. Pregnant women attending healthcare facilities in Ambon City between June and October 2024 were recruited. Ethical approval was obtained from the Ethics Committee Review Board of the Faculty of Medicine, Universitas Pattimura, with reference number 109/FK-KOM.ETIK/VIII/2024.

This study categorized participants into four groups based on the following inclusion criteria:

1. Pregnant Women with Preeclampsia Consuming Marine Fish.

- (a) Pregnant women with a gestational age of 20 to ≤ 34 weeks determined by the first day of the last menstrual period or crown-rump length (CRL) measurements at 11–14 weeks of gestation, who agreed to participate in the study.
- (b) All parity levels.
- (c) Maternal age < 40 years.
- (d) Diagnosed with preeclampsia by a clinician.
- (e) Consumption of marine fish at a minimum of four pieces per day or at least one medium-sized fish daily.

2. Pregnant Women Without Preeclampsia Consuming Marine Fish.

- Pregnant women with a gestational age of 20 to ≤ 34 weeks determined by the first day of the last menstrual period or CRL measurements at 11–14 weeks of gestation, who agreed to participate in the study.
- All parity levels.
- Maternal age < 40 years.
- Consumption of marine fish at a minimum of four pieces per day or at least one medium-sized fish daily.

3. Pregnant Women with Preeclampsia Consuming Less Marine Fish.

- Pregnant women with a gestational age of 20 to ≤ 34 weeks determined by the first day of the last menstrual period or CRL measurements at 11–14 weeks of gestation, who agreed to participate in the study.
- All parity levels.
- Maternal age < 40 years.
- Diagnosed with preeclampsia by a clinician.
- Consumption of marine fish less than four pieces per day or less than one medium-sized fish daily.

4. Pregnant Women Without Preeclampsia Consuming Less Marine Fish.

- Pregnant women with a gestational age of 20 to ≤ 34 weeks determined by the first day of the last menstrual period or CRL measurements at 11–14 weeks of gestation, who agreed to participate in the study.
- All parity levels.
- Maternal age < 40 years.
- Consumption of marine fish less than four pieces per day or less than one medium-sized fish daily.

### Exclusion criteria

1. Pregnant women with a medical history of chronic hypertension, hyperthyroidism, type 1 or type 2 diabetes mellitus, thrombophilia, autoimmune diseases (e.g., systemic lupus erythematosus [SLE], antiphospholipid syndrome [APS], unexplained recurrent miscarriage), kidney disease, tuberculosis, or other chronic illnesses diagnosed by the respective medical department.
2. Multifetal pregnancies confirmed through ultrasound examination.
3. Obesity (Body Mass Index [BMI] > 30 kg/m²) to reduce confounding effect of obesity on preeclampsia risk.

### Study groups

Participants were categorized into four groups based on micronutrient status and fish intake. Age categories were defined as < 20 years, 20–35 years, and > 35 years, with < 20 and > 35 considered high-risk pregnancy groups. Gestational age was classified as 20–28 weeks (including 28 weeks) and 29–34 weeks (including 29 weeks). Recruitment was restricted to 20–34 weeks of gestation, as this range provides greater diagnostic stability for preeclampsia biomarkers.

### Dietary assessment

Dietary intake was measured using a Food Frequency Questionnaire (FFQ) adapted from the Indonesian National FFQ and pilot tested locally to ensure cultural relevance and comprehension. While the FFQ was adapted for the Ambonese population, it was not fully validated against biomarkers; this limitation is acknowledged. Fish intake was categorized based on prior regional dietary surveys, where ≥ 4 pieces/day (approximately 480 g/day, with one piece equivalent to ~ 120 g) reflects high habitual consumption.

### Sampling and laboratory procedures

No specific preparation is required for patients undergoing calcium testing. The specimen can be collected at any time, with no restrictions on timing. The required sample is serum, with a volume of 100–200 µL, and no preservatives should be used. Proper handling of the sample is crucial; serum and plasma must be separated promptly according to standard specimen handling protocols, as extended contact with the clot can lower calcium levels. The sample should be collected in plain tubes with a capacity of 3, 4, or 5 mL. There are no special requirements for transport media. However, samples with hemolysis, lipemic, or icteric properties may not be outright rejected, while re-frozen specimens are subject to absolute rejection.

For zinc testing, patient preparation involves avoiding blood collection within 96 h of exposure to contrast agents containing gadolinium, iodine, or barium, as these can interfere with metal analysis. Blood samples can be collected at any time, but iodine-based antiseptics such as Betadine should not be used during collection. The required sample is serum collected in a trace element clot activator tube, with a volume of 250–750 µL, and no preservatives are needed. Samples should be allowed to clot for 30 min before centrifugation, and serum must be separated from blood cells within two hours of collection to ensure accuracy. The sample should be placed in a 9 mL trace element tube, ensuring the container is free of metal contamination. There are no specific transport media requirements, but specimens with hemolysis are rejected outright, while lipemic and icteric samples are conditionally accepted. Re-frozen specimens are strictly rejected.

### Statistical analysis

The subsequent analysis aimed to describe the dependent and independent variables, facilitating a deeper understanding and aiding in more comprehensive analyses. Descriptive analysis was employed to identify the characteristics of the research subjects that constitute the study sample. Data proportions for each variable were presented descriptively, utilizing both descriptive statistics and hypothesis testing. Numerical data, such as patient age, were summarized using measures like mean, standard deviation, median, and range. Categorical data reflecting sample characteristics were encoded and presented as frequency distributions and percentages.

The analysis was conducted based on the research problem and the nature of the data. For numerical data, normality is assessed using the Shapiro-Wilk test for datasets with fewer than 50 observations, or the Kolmogorov-Smirnov test for larger datasets. These tests determined whether the data follow a normal distribution. Statistical tests were then applied according to the research objectives and hypotheses. For comparing the characteristics of two groups, an independent t-test was used for normally distributed data, while the Mann-Whitney U test served as an alternative for non-normally distributed data. Categorical data were analyzed using the Chi-square test if its assumptions were met, or alternatively, the Fisher’s Exact test for 2 × 2 tables and the Kolmogorov-Smirnov test for tables with other dimensions. The Chi-square test required that no more than 20% of expected values in the table fall below 5. For this case-control study, Odds Ratio (OR) values were calculated to quantify the strength of causal relationships. Statistical significance was determined by a p-value threshold of ≤ 0.05, with values above 0.05 indicating non-significant results. Data were recorded on dedicated forms and processed using SPSS version 26.0 for Windows.

## Results

The study included 120 participants divided equally into preeclamptic and non-preeclamptic groups.

**Table 1:** Comparison of characteristics of research subjects between preeclampsia and Non-Preeclampsia groups based on demographic data

Variables	Groups		*P*-Value
	Preeclampsia	Non-Preeclampsia	
	*n* = 60	*n* = 60	
**Maternal Age**			0.01*
< 20 years	1(1.7%)	3(5.0%)	
20–35 years	40(66.7%)	51(85.0%)	
> 35 years	19(31.7%)	6(10.0%)	
**Usia Kehamilan**			0.01**
20–28 weeks	3(5.0%)	25(41.7%)	
29–34 weeks	57(95.0%)	35(58.3%)	
**Parity**			**0.13**
0–1	36(60.0%)	44(73.3%)	
2–4	23(38.3%)	15(25.0%)	
$\:\ge\:$5	1(1.7%)	1(1.7%)	
**Education**			**0.68**
< 9 years	4(6.7%)	2(3.3%)	
$\:\ge\:$9 years	56(93.3%)	58(96.7%)	
**Employment Status (Yes/No)**			**0.53**
Yes	14(23.3%)	17(28.3%)	
No	46(76.7%)	43(71.7%)	

Note: Ordinal data p-values ​​are tested using the Mann Whitney test. Categorical data p-values ​​are calculated based on the Chi-Square test with alternative Kolmogorov-Smirnov and Exact Fisher tests if the requirements of Chi-Square are not met. Significance values ​​are based on p-values ​​<0.05. * *p* < 0.05

Table 1 compares the demographic characteristics of research subjects between the preeclampsia group and the non-preeclampsia group. The data indicate significant differences in maternal age and gestational age (*p* < 0.05). However, there are no statistically significant differences in parity, education level, or employment status between the two groups (*p* > 0.05). These findings suggest that, apart from maternal age and gestational age, the two groups are homogeneous and suitable for further hypothesis testing.

**Table 2:** Comparison of daily calcium consumption and daily zinc consumption based on food frequency questionnaire (FFQ) in the preeclampsia group and the Non-Preeclampsia group

Variables	Groups		*P*-Value
	Preeclampsia	Non-Preeclampsia	
	*N* = 60	*N* = 60	
**Daily Calcium Consumption**			**0.33**
Deficient	43(71.7%)	38(63.3%)	
Sufficient	17(28.3%)	22(36.7%)	
**Daily Zinc Consumption**			
Deficient	47(78.3%)	43(71.7%)	**0.39**
Sufficient	13(21.7%)	17(28.3%)	

* Note: Ordinal data p-values ​​are tested using the Mann Whitney test. Categorical data p-values ​​are calculated based on the Chi-Square test with alternative Kolmogorov Smirnov and Exact Fisher tests if the requirements of Chi-Square are not met. Significance values ​​are based on p-values ​​<0.05. * *p* < 0.05

Table 2 illustrates the daily calcium and zinc intake among the research subjects. There are no statistically significant differences in daily calcium and zinc intake between the preeclampsia and non-preeclampsia groups (*p* > 0.05). While some studies suggest an association between low calcium intake and the incidence of preeclampsia, this study did not find such a relationship, possibly due to differences in dietary habits and individual nutritional status.

**Table 3:** Comparison of fish types and fish processing methods in the preeclampsia group and without preeclampsia

Variables	Groups		*P*-Value
	Preeclampsia	Non-Preeclampsia	
	*N* = 30	*N* = 30	
**Fish Species**			**0.39**
Mackarel/lema fish	1(3.3%)	3(10.0%)	
Tongue/komu fish	13.3%)	6(20.0%)	
Scad/momar fish	17(56.7%)	11(36.7%)	
Tuna/tatihu fish	1(3.3%)	1(3.3%)	
Skipjack tuna	3(10.0%)	1(3.3%)	
Selar/ kawalinya fish	7(23.3%)	8(26.7%)	
**Processing Methos**			**0.95**
Fried	19(63.3%)	23(76.7%)	
Boiled	2(6.7%)	2(6.7%)	
Grilled	8(26.7%)	1(3.3%)	
Roasted	1(3.3%)	4(13.3%)	

Note: Descriptive analysis * *p* < 0.05

Based on Table 3, in the preeclampsia group, the data of mothers who consumed the most types of fish were mackerel/momar fish as many as 17 or 56.7%. with the most common processing method being fried as many as 19 or 63.3%. In the group without preeclampsia, the data of patients who consumed the most types of fish were mackerel/momar fish as many as 11 or 36.7%. with the most common processing method being fried as many as 23 or 76.7%. Analysis of categorical data in the table above, was tested using the Kolmogorov Smirnov statistical test. The results of the statistical test in the research group above obtained information on the p value on the type of fish and fish processing method variables greater than 0.05 (P-value > 0.05) which means it is not significant or not statistically significant. Thus, it can be explained that there is no statistically significant difference between the types of fish and fish processing methods in the preeclampsia and non-preeclampsia groups.

**Table 4:** Comparison of calcium and zinc levels in pregnant women with preeclampsia and without preeclampsia who consume sea fish

Variables	Groups		OR CI 95%	P-Value
	Preeclampsia	Non-Preeclampsia		
	*N* = 60	*N* = 60		
**Calcium Levels**			**5.035**	0.01**
Low	41(68.3%)	18(30.0%)	**(2.319–10.930)**	
Normal	19(31.7%)	42(70.0%)		
**Zinc Levels**				**0.053**
Low	56(93.3%)	49(81.7%)		
Normal	4(6.7%)	11(18.3%)		

*Notes: For ordinal data, the p value was tested using the Mann Whitney test. Categorical data p value is calculated based on the Chi-Square test with alternative Kolmogorov Smirnov and Exact Fisher tests if the requirements of Chi-Square are not met. The significance value is based on the p value < 0.05. *CI: Confidence Interval. * *p* < 0.05

Table 4 shows no statistically significant difference in zinc levels between pregnant women with and without preeclampsia who consumed sea fish. However, the borderline significance of zinc has been highlighted in the discussion, reflecting its possible role despite limited statistical power. Effect sizes for key findings have also been reported to aid interpretation beyond p-values.

Based on odds ratio analysis, women with low calcium levels had a 5.035-fold higher likelihood of developing preeclampsia compared to those with normal calcium levels (95% CI: 2.319–10.930). Similarly, women with low zinc levels had a 3.143-fold higher likelihood of preeclampsia compared to those with normal zinc levels (95% CI: 0.940–10.507). While the association for calcium was statistically significant, the zinc finding remained marginal and should be interpreted with caution. All tables have been revised and presented fully in English, with Indonesian terms removed for clarity.

**Table 5:** Comparison of calcium and zinc levels of pregnant women in the group with preeclampsia based on history of consuming sea fish

Variables	Sea Fish Consumption		OR CI 95%	*P*-Value
	< 4	≥ 4		
	*N* = 30	*N* = 30		
**Calcium Levels (Preeclampsia)**			**10.286** **(2.557–41.372)**	0.01*
Low	27(90.0%)	14(46.7%)		
Normal	3(10.0%)	16(53.3%)		
**Zinc Levels (Preeclampsia)**				**0.61**
Low	27(90.0%)	29(96.7%)		
Normal	3(10.0%)	1(3.3%)		

Notes: For ordinal data, the p value was tested using the Mann Whitney test. Categorical data p value is calculated based on the Chi-Square test with alternative Kolmogorov Smirnov and Exact Fisher tests if the requirements of Chi-Square are not met. The significance value is based on the p value < 0.05. *CI: Confidence Interval. * *p* < 0.05

**Table 6:** Comparison of calcium and zinc levels of pregnant women in the group without preeclampsia based on history of consuming sea fish

Variables	Sea Fish Consumption		OR CI 95%	*P*-Value
	< 4	≥ 4		
	*N* = 30	*N* = 30		
**Calcium Levels (Non-Preeclampsia)**			**5.688** **(1.591–20.330)**	0.01*
Low	14(46.7%)	4(13.3%)		
Normal	16(53.3%)	26(86.7%)		
**Zinc Levels (Non-Preeclampsia)**				**0.74**
Low	24(80.0%)	25(83.3%)		
Normal	6(20.0%)	5(16.7%)		

Notes: For ordinal data, the p value was tested using the Mann Whitney test. Categorical data p value is calculated based on the Chi-Square test with alternative Kolmogorov Smirnov and Exact Fisher tests if the requirements of Chi-Square are not met. The significance value is based on the p value < 0.05. *CI: Confidence Interval. * *p* < 0.05

Tables 5 and 6 shows based on the odds ratio value above, it can be concluded that the possibility of preeclampsia patients with low calcium levels who consume sea fish < 4 pieces per day is 10,286 times compared to normal calcium levels who consume sea fish ≥4 pieces per day. With a confidence interval of (2,557–41,372). Meanwhile, the possibility of preeclampsia patients with low zinc levels consuming < 4 pieces of sea fish per day is 0.310 times compared to preeclampsia patients with normal zinc levels who consume ≥4 pieces of sea fish per day, with a confidence interval of (0.020–3.168), while patients without preeclampsia with low calcium levels who consume sea fish < 4 pieces per day is 5,688 times compared to patients without preeclampsia with normal calcium levels who consume sea fish ≥4 pieces per day, with a confidence interval of (1,591–20,330). Meanwhile, the possibility of patients without preeclampsia with low zinc levels who eat fish < 4 pieces per day is 0,800 times compared to patients without preeclampsia with normal zinc levels who consume sea fish ≥4 pieces per day, with a confidence interval of (0.215–2.972).

**Table 7:** Multivariate analysis

Variables		B	S.E.	P-Value	OR	OR CI 95%	
						Lower	Upper
Early Model	1. Maternal Age	-0.833	0.509	0.101	0.435	0.160	1.178
	2. Gestational Age	-2.370	0.662	0.01*	0.093	0.026	0.342
	3. Calcium Levels	1.426	0.449	0.01*	4.161	1.726	10.034
Late Model	1. Maternal Age	-2.418	0.659	0.01*	0.089	0.025	0.324
	2. Calcium Levels	1.597	0.437	0.01*	4.938	2.097	11.629

Multivariable logistic regression was applied to examine the association of maternal age, gestational age, and calcium levels with preeclampsia. Variables with *p* < 0.25 in univariate analysis were included in the initial (early) model. A backward elimination process was then used to derive the final (late) model, retaining only variables that remained statistically significant. Odds ratios (OR) and 95% confidence intervals (CI) were calculated, and a p-value < 0.05 was considered statistically significant.* *p* < 0.05

After bivariate analysis, multivariate analysis of the relationship between several independent variables and the incidence of preeclampsia was continued. The independent variables included in the model are variables in the bivariate analysis that have a value of less than 0.05, namely maternal age, gestational age and calcium levels. The aim is to analyze which of the three independent variables most dominantly influences the incidence of preeclampsia. The results of the multivariate analysis in the initial model did not have all p-values ​​of variables that were smaller than 0.05 (*p* < 0.05). This shows that simultaneously the overall maternal age, gestational age and calcium levels do not affect the occurrence of preeclampsia. From the Table 7 above based on the final model which can be seen from the three initial model variables after going through two stages, the best model is in the final model, namely the p-value that is significant and most influences the incidence of preeclampsia, namely the variables of gestational age and calcium levels. So it can be concluded that statistically only the risk factors of gestational age and calcium levels are strongly related in predicting the incidence of preeclampsia. Based on the odds ratio value in the final model, it can be concluded that pregnant women with low calcium levels are at risk of experiencing preeclampsia 4,938 times, while those aged 29–34 years are only at risk 0,089 times.

Regarding dietary patterns, no statistically significant differences were observed between the types of fish consumed or the preparation methods (*p* > 0.05). However, the predominance of fried fish consumption in both groups may have influenced nutrient bioavailability, potentially attenuating the protective effects of zinc and calcium intake. Effect size estimates indicated that women with low calcium levels had a 5.04-fold higher likelihood of developing preeclampsia (95% CI: 2.32–10.93), while those with low zinc levels had a 3.14-fold increased risk (95% CI: 0.94–10.51), although the latter did not reach conventional statistical significance.

## Discussion

Preeclampsia is a leading cause of maternal morbidity and mortality, primarily due to pregnancy-related stressors impacting cardiovascular and metabolic systems. Maternal nutrition is pivotal in supporting optimal placental and fetal development, and dietary patterns may play a preventive role against preeclampsia. Research has identified deficiencies in micronutrients such as calcium and zinc as significant risk factors for preeclampsia. These essential trace elements contribute to critical physiological processes, including cellular metabolism, antioxidant defenses, anti-inflammatory mechanisms, enzymatic activities, gene regulation, and protein synthesis. Insufficient calcium and zinc levels during pregnancy are associated with complications such as hypertension and preeclampsia. Moreover, inflammation induced by nutrient deficiencies may impair placental function, further exacerbating the risk [15, 16].

The study explored the relationship between calcium and zinc levels and preeclampsia among pregnant women consuming marine fish in Ambon City. Statistical analysis revealed that maternal age and gestational age were significantly associated with preeclampsia, while parity, education level, and employment status showed no significant differences between groups. Women aged 20–35 years, often experiencing their first pregnancy, exhibited higher preeclampsia prevalence, likely due to genetic and lifestyle factors. Gestational age between 29 and 34 weeks was the most common period for preeclampsia onset, aligning with studies linking early-onset preeclampsia to inadequate uterine artery remodeling and systemic inflammation [16–18]. Morevoer, calcium levels showed a significant difference between preeclampsia and non-preeclampsia groups, with lower levels correlated with increased risk. Conversely, zinc levels, though more frequently low in preeclampsia cases, did not exhibit significant differences.

Calcium deficiency disrupts physiological functions, leading to endothelial dysfunction, activation of the Renin-Angiotensin-Aldosterone System (RAAS), insulin resistance, oxidative stress, inflammation, and platelet dysfunction, all of which elevate preeclampsia risk. Zinc deficiency, on the other hand, compromises antioxidant capacity by reducing superoxide dismutase (SOD) activity, disrupting the oxidative-antioxidant balance essential in mitigating preeclampsia. Regular intake of calcium and zinc through supplementation or diet is therefore recommended during pregnancy. In Indonesia, fish is a primary dietary source of these minerals, particularly in regions like Maluku, which has the highest fish consumption nationally. High fish consumption should ideally mitigate preeclampsia risks by fulfilling calcium and zinc requirements [19–21].

The study highlighted the protective role of calcium in vascular homeostasis, endothelial function, and inflammation regulation. However, it noted inconsistencies in zinc’s role, suggesting potential geographic or methodological factors. Marine fish consumption positively correlated with improved calcium and zinc levels, emphasizing the importance of dietary interventions in mitigating preeclampsia risk [23, 24]. This study highlights the critical role of calcium in preventing preeclampsia among pregnant women consuming seafood. Consistent with prior research, calcium deficiency is implicated in vascular dysfunction and oxidative stress, both hallmarks of preeclampsia.

Biological plausibility supports these findings. Calcium deficiency disrupts physiological functions, leading to endothelial dysfunction, activation of the Renin-Angiotensin-Aldosterone System (RAAS), insulin resistance, oxidative stress, inflammation, and platelet dysfunction, all of which elevate preeclampsia risk. Importantly, calcium absorption and bioavailability are influenced by dietary sources; fish provides a highly bioavailable form of calcium compared to certain plant-based sources, which are often inhibited by phytates and oxalates. Zinc deficiency, on the other hand, compromises antioxidant capacity by reducing superoxide dismutase (SOD) activity, disrupting the oxidative-antioxidant balance essential in mitigating preeclampsia. It is biologically plausible that zinc plays a modulatory role in endothelial and placental function, but the absence of statistical significance in this study may reflect insufficient power, heterogeneity in dietary intake, or unmeasured confounding [16–24].

Marine fish consumption was expected to provide adequate calcium and zinc intake; however, preparation methods may reduce their bioavailability. Frying, the most common method observed in this cohort, has been shown to degrade heat-sensitive nutrients and increase oxidative lipid byproducts, potentially offsetting the benefits of micronutrient intake. In contrast, boiling and grilling may better preserve micronutrient content. This highlights the need for future studies to consider food processing as a modifier of nutritional effects [23, 24].

Beyond biological mechanisms, socioeconomic factors and supplement use represent important limitations. Access to prenatal supplements, affordability of diverse diets, and differences in healthcare access may confound associations. As this study relied on an FFQ adapted from the Indonesian National FFQ but not fully validated locally, dietary intake estimates may also have measurement limitations.

From a public health perspective, the findings strengthen the case for comprehensive antenatal nutrition education and supplementation strategies. Calcium supplementation, already recommended by the WHO for populations with low dietary intake, should be emphasized in maternal health programs. Zinc, despite showing borderline significance, warrants continued attention given its biological role and consistent associations in prior meta-analyses. At the policy level, integrating locally available resources such as fish into maternal nutrition education can support dietary adequacy, while also addressing preparation methods to maximize nutrient retention.

The results of this study are expected to serve as a valuable reference for future research to further verify the role of natural resources, particularly fish, as a source of animal protein rich in micronutrients such as calcium, zinc, and other substances that contribute to the prevention of preeclampsia and fetal development. This is also related to raising public awareness about the importance of consuming fish regularly during pregnancy, especially in areas abundant in natural resources, such as Ambon City and its surroundings.

## Conclusion

In Ambon City, pregnant women with preeclampsia have lower calcium levels compared to those without preeclampsia, despite consuming marine fish. However, zinc levels do not show a significant difference between the two groups. Among pregnant women with preeclampsia, those who consume less than four medium-sized pieces of marine fish per day have lower calcium levels than those who consume four or more pieces daily, while their zinc levels remain unchanged regardless of fish intake. Similarly, in pregnant women without preeclampsia, consuming at least four medium-sized pieces of marine fish per day is associated with more normal calcium levels compared to those who consume less. However, zinc levels in this group do not differ based on the amount of fish consumed.

### Limitations

This study was limited by its reliance on self-reported dietary intake, which may have introduced recall bias. Specifically, maternal recall restricted the accuracy of data on fish consumption and preparation methods, limiting the ability to assess how different cooking practices may have affected micronutrient bioavailability. Another limitation was the lack of detailed information on supplement use or other dietary sources of calcium and zinc, which could have influenced serum levels and confounded associations. Additionally, the absence of a statistically significant association between zinc levels and preeclampsia requires further investigation, particularly in larger cohorts with more comprehensive dietary assessments. Future research should address these gaps to strengthen understanding of the role of micronutrients in preeclampsia prevention.

## Data Availability

The authors declare that the personal data from any patients involved in this study will not be shared based on patients’ confidentialities.

## Abbreviations

DNA: Deoxyribonucleic acid
NO: Nitrit Oxide
PE: Preeclampsia
RAAS: Renin-Angiotensin-Aldosterone System
SOD: Superoxide Dismutase
WHO: World Health Organization
